# Socioeconomic inequality in self-reported unmet need for oral health services in adults aged 50 years and over in China, Ghana, and India

**DOI:** 10.1186/s12939-018-0812-2

**Published:** 2018-07-11

**Authors:** Alexander Kailembo, Raman Preet, Jennifer Stewart Williams

**Affiliations:** 1 Oral Health Programme, Non-Communicable Diseases Cluster, World Health Organization, PO Box 9292, Dar Es Salaam, Tanzania; 2 Umeå International School of Public Health, Unit of Epidemiology and Global Health, Department of Public Health and Clinical Medicine, Faculty of Medicine, Umeå University, SE-90185 Umeå, Sweden; 3 Research Centre for Generational Health and Ageing, Faculty of Health, University of Newcastle, New Lambton Heights, NSW 2305 Australia

**Keywords:** Dental, Inequalities, Low-and middle-income countries, Ageing, Socioeconomic status, Access to care, Oral health policy

## Abstract

**Background:**

The 2015 Global Burden of Disease Study estimated that oral conditions affect 3.5 billion people worldwide with a higher burden among older adults and those who are socially and economically disadvantaged. Studies of inequalities in the use of oral health services by those in need have been conducted in high-income countries but evidence from low- and middle-income countries (LMICs) is limited. This study measures and describes socioeconomic inequality in self-reported unmet need for oral health services in adults aged 50 years and over, in China, Ghana and India.

**Methods:**

A cross-sectional analysis of national survey data from the WHO SAGE Wave 1 (2007–2010) was conducted. Study samples in China (*n* = 1591), Ghana (*n* = 425) and India (*n* = 1307) were conditioned on self-reported need for oral health services in the previous 12 months. The binary dependent variable, unmet need for oral health services, was derived from questions about self-reported need and service use. Prevalence was estimated by country. Unmet need was measured and compared in terms of relative levels of education and household wealth. The methods were logistic regression and the relative index of inequality (RII). Models were adjusted for age, sex, area of residence, marital status, work status and self-rated health.

**Results:**

The prevalence of unmet need was 60, 80, and 62% in China, Ghana and India respectively. The adjusted RII for education was statistically significant for China (1.5, 95% CI:1.2–1.9), Ghana (1.4, 95% CI: 1.1–1.7), and India (1.5, 95% CI:1.2–2.0), whereas the adjusted RII for wealth was significant only in Ghana (1.3, 95% CI:1.1–1.6). Male sex was significantly associated with self-reported unmet need for oral health services in India.

**Conclusions:**

Given rapid population ageing, further evidence of socioeconomic inequalities in unmet need for oral health services by older adults in LMICs is needed to inform policies to mitigate inequalities in the availability of oral health services. Oral health is a universal public health issue requiring attention and action on multiple levels and across the public private divide.

## Background

The FDI (fédération dentaire internationale) or World Dental Federation defined oral health as a multifaceted health state and a fundamental component of overall health and mental well-being, reflected by the physiological, social, and psychological attributes which are essential for quality of life [1]. Oral diseases are among the highest prevalent health conditions in the world and they pose major public health challenges. The 2015 Global Burden of Disease Study estimated that oral diseases and conditions (untreated caries, severe periodontitis, and edentulism) affect 3.5 billion people worldwide [2]. Disability adjusted life-years due to oral conditions increased by 64% from 10.3 to 16.9 million between 1990 and 2015, largely as a consequence of population growth and ageing [2].

In 2015, the global proportion of people aged 65 years and over was 8.5%. By 2050, this segment of the population will comprise about 16.7% of the estimated total global population of 9.4 billion people [3]. In sheer numbers this translates to an average annual increase of 27.1 million people aged 65 years and over between 2015 and 2050 [3]. With greater numbers of people living to older age, the need for oral health services will also increase. Because of the cumulative effect of social and economic factors that impact on access to health care and the lack of priority given to oral health in general, older adults may have limited resources and opportunities to meet their ongoing oral health care needs [4].

Inequalities in oral disease are of global public health concern [5]. Oral health is distributed unequally between rich and poor countries with lowest coverage in low-income countries (LICs) [6]. In the World Health Surveys (2002–2004) the proportion of adults aged 65–74 who reported teeth or mouth problems was 40% in LICs compared with 30% in high-income countries (HICs), and the proportion of people who reported receiving oral health care was 30% in LICs in contrast to 75% in HICs [5]. Edentulism is a marker of poor oral health in low-and middle-income countries (LMICs) where service coverage is low. An analyses of cross sectional data from the World Health Organization (WHO) Study on AGEing and adult health (SAGE) Wave 1 demonstrated that people aged 50 and over were more likely to be edentulous in India than in China [7, 8]. Oral health service use is influenced by the availability of trained personnel and specialised resources. In 2015 the dentist-to-population ratio was approximately 1:150,000 in countries in the African region, compared with 1: 2000 in HICs [9–11].

There are inequalities in oral health between different social groupings in rich and poor countries. A multi-country study of adults aged 50 years and over in fourteen European countries reported higher rates of oral health service use among high- compared with low-income population groups [12]. Socioeconomic inequalities in oral health service have been reported for HICs such as Australia [13], Denmark [14], Sweden [15] and the United States [16]. Research in Nigeria [17] and China [18] demonstrated that people of lower socioeconomic position had lower oral health service use compared with those who were relatively socioeconomically advantaged. More international studies of oral health use are needed, particularly in LMICs where dental infrastructure is limited and health burdens from oral diseases are rising.

The same social determinants that apply to general health also apply to oral health whereby the burden is higher among those who, for example, have less wealth and education, and experience poor housing and working conditions. Older people without access to adequate social protection are a vulnerable segment of the population [19–21] and social and economic factors, such as lower education and household wealth, are associated with the use of dental services [7, 8].

Unmet need is one of the indicators used to monitor inequalities in access to and use of health services. This can be assessed by asking survey respondents whether there was a time (usually in the prior 12 months) when they needed health services but did not receive them [22, 23]. Unmet need, so defined, is captured in international studies such as the Survey on Health, Ageing and Retirement, the European Union Survey of Income and Living Conditions, and WHO-SAGE [22, 24]. This study investigates socioeconomic patterning of self-reported need for oral health services in China, Ghana and India – three LMICs at different stages of social and economic development.

China is a rapidly developing country of 1.4 billion people, a substantial proportion of whom are already beyond mid-age. In 2017 the proportion of China’s population aged 60 and over was 16% [25]. Since the late 1970s China has undergone rapid economic development which has led to increases in per capita income, declines in average poverty rates and better public health care. Yet despite these aggregate improvements, inequalities in wealth and health have widened in recent decades and there are well documented social gradients in health and access to care [26, 27]. Many poor people are living longer but experiencing chronic illnesses [28–30]. Dental caries and periodontal diseases are increasing in prevalence [31]. Although China’s reform agenda includes universal insurance for preventive and curative oral services [32] and increased training positions for dental professionals, the population remains underserved by oral health services [30].

Ghana is a lower middle-income country on the west coast of the African continent. In 2017 the population of Ghana was 28.8 million [25]. People are living longer, and although infectious diseases still contribute the largest share of the country’s disease burden, chronic diseases are increasing in prevalence [33, 34]. Social security protection, health insurance and health care resources are deficient, particularly for the aged. Ghana has a ratio of one dentist per 104,000 population [35] which compares with about one to 2000 in many HICs. In Ghana 75% of working dentists are located in urban areas and consequently rural areas are underserved [35].

With a population of 1.3 billion people, India is the world’s second most populous country, after China. Economic growth has been relatively slower than China’s, and poverty rates are higher. Health care is mostly delivered through a large unregulated private sector [36]. Unlike China [29], India still faces a double burden of both communicable and non-communicable diseases (NCDs) [36]. Although oral health policy was introduced into India’s National Health Policy in the 1990s, progress has been slow [37]. The poor do not use dental services to the same extent as the rich and affordability is a barrier for use [37, 38].

Poor oral health can have a deleterious impact on older adults’ quality of life. Oral diseases can be chronic, and they have many risk factors in common with other NCDs [19, 39]. People with greater resources, generally have relatively more opportunities to access professional care and treatment to meet their oral health needs [5, 9]. Yet most of this evidence originates from studies conducted in better resourced HICs [40]. Greater understanding of these issues is important for designing and implementing targeted policies to improve the oral health of populations in LMICs.

The aim of this study is to measure and describe education and wealth socioeconomic inequalities in self-reported unmet need for oral health services among adults, aged 50 and over, in China, Ghana and India. Based on our reading of the literature, we hypothesise that inequalities in unmet need will follow a similar pattern to that of inequalities in oral health whereby those with lower wealth and education have more unmet need for oral health services. Considering country income levels, we also hypothesise that unmet need for oral health care is highest in Ghana, the poorest of the three countries, and lowest in China, the richest of these three countries.

## Methods

### Data source

The data source, WHO SAGE Wave 1 (2007–2010), is a longitudinal study of nationally representative samples of adults aged 50 years and over in China, Ghana, India, Mexico, Russia and South Africa. In this study we draw on the cross-sectional results of WHO SAGE Wave 1 national surveys implemented between 2007 and 2010 in China, Ghana and India. Data were obtained from self-reported responses to validated structured household and individual questionnaires.

WHO-SAGE Wave 1 used a stratified random sampling frame. Sampling weights were applied to adjust for age and sex distributions, urban and rural localities and non-response. In China the sample was stratified into eight nationally representative provinces. The Ghana sample was stratified by administrative region and urban or rural locality, giving eighteen nationally representative strata. In India states were selected in accordance with geographic location and level of development. The sample in India was stratified by state and urban or rural locality giving 12 nationally representative strata [41].

Data collection in Ghana and India was through face-to-face paper and pencil interviews. In China 50% of the data were collected by face-to-face computer assisted personal interviews and 50% by face-to-face paper and pencil interviews [41]. A standardised questionnaire was used in all countries. Questionnaires were translated into local languages, validated and then back-translated. Interviews were conducted by trained interviewers in culturally appropriate settings. WHO protocols were closely monitored throughout data collection [42].

WHO-SAGE country data sets and sampling weights, are in the public domain. Further details of WHO-SAGE are provided elsewhere [43].

### Study population

WHO SAGE Wave 1 included 47,443 respondents aged 18 and over. This analysis covers samples drawn from SAGE respondents aged 50 and above in China (*n* = 15,050), Ghana (*n* = 5565) and India (*n* = 12,198). Response rates were 93, 80, and 68% in respectively [44]. Respondents less than 50 years of age in China (1636), Ghana (805) and India (4670) were excluded as were those who did not complete the WHO-SAGE individual questionnaire - China (*n* = 237), Ghana (463) and India (968).

Respondents who completed the surveys and were aged 50 years and over were: 13,177 in China, 4297 in Ghana and 6560 in India. The country samples were further conditioned on self-reported need for oral health services elicited from two key questions outlined in the section below. Only those respondents who expressed a need for oral health services were included giving 1607 in the China sample, 431 in the Ghana sample and 1316 in the India sample. A further 31 records were excluded due to missing data on key variables (China 16, Ghana 6 and India 9).

**Fig. 1:**
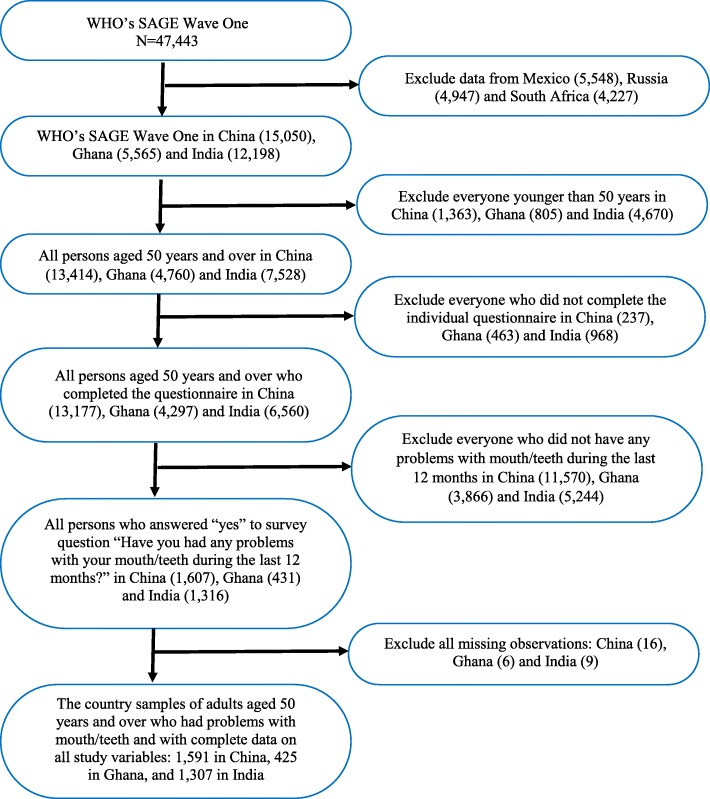
Flowchart of participants in the study. Data source – WHO Study on global AGEing and adult health (SAGE) Wave 1 (2007–2010)

The Mexican (*n* = 5548) Russian (*n* = 4947) and South African (*n* = 4227) samples were not analysed here because of high percentages of missing data on the study variables (52 and 13% in Mexico and Russia respectively) and small sample sizes after conditioning for self-reported need for oral health (e.g. 81 in South Africa). See Fig. 1.

### Dependent variable

The dependent variable, unmet need for oral health services, was derived from self-reported answers to two survey questions: 1) “Have you had any problems with your mouth/teeth during the last 12 months?” and 2) “Have you received any medication or treatment from a dentist or any other oral health specialist during the last two weeks or 12 months?” Only respondents who answered “yes” to the first question were considered for the second question. A binary indicator variable (unmet need) was derived from answers to the second question - those who responded “yes” were classified as having no unmet need for oral health services in the prior 12 months and those who responded “no” were classified as having unmet need in the prior 12 months.

### Socioeconomic variables

Two categorical independent variables – household wealth and education – were the socioeconomic measures used to quantify inequality in self-reported unmet need for oral health services.

The household wealth variable was based on information on ownership of household assets reported in the WHO-SAGE household questionnaire. This included ownership of durable goods (e.g. car, television, radio, and refrigerator), household characteristics (e.g. type of windows, roof and floor material), and access to basic services (e.g. source of water and electricity). Principal component analysis was used to generate weights for household assets [45]. Wealth quintiles were produced with quintile one representing the lowest wealth and quintile five the highest. In this study, the wealth quintiles were recoded with quintile one representing the highest wealth and quintile five the lowest.

The education variable was derived from two survey questions “Have you ever been to school?” and “What is your highest level of education completed?” The variable indicated the highest reported level of education and was categorized in four groups; university or higher, secondary, primary, and no schooling.

### Covariates

Covariates were: sex (male or female); age categorized as 50–59 years, 60–69 years, 70–79 years, and 80 years and over; area of residence (urban or rural); marital status (never married versus married or cohabiting versus widowed, divorced or separated); work status (never worked versus currently working versus currently not working), and self-rated health (good versus moderate versus bad).

### Statistical analysis

Only records with complete data on all study variables were included in these analyses. Survey sampling weights were used in all analyses. Descriptions of the country samples are given by absolute numbers and weighted percentages. The prevalence of unmet need was estimated with 95% confidence intervals (CIs) to allow statistical comparison between countries.

Logistic regressions were used to measure inequalities in unmet need. Univariable models were derived separately for household wealth and education. Multivariable models, controlling for age, sex, area of residence, marital status, work status, and self-rated health, were conducted separately for household wealth and education to adjust for potential confounding. Odds ratios (ORs), 95% CIs and *p*-values are reported.

The relative index of inequality (RII) was used to separately measure inequalities in unmet need for oral health services in terms of household wealth and education. The RII is a summary measure which quantifies relative socioeconomic inequalities in an outcome by taking into account the size of the population across ordered socioeconomic categories [46]. Here we have two RIIs – one for household wealth and one for education. The RII is defined as the ratio of the estimated prevalence of unmet need (for oral health services) between the poorest and wealthiest or between the least educated and the most educated. If, for example, the RII for wealth is 1.6 and statistically significant this means that the estimated prevalence of unmet need is 1.6 times higher among the poorest group compared with wealthiest group. If the RII is close to one (or not statistically significant) then we can say that there is no evidence of socioeconomic inequality. If the score is less than one and significant, for example 0.5, then in this example, the unmet need would be 50% lower in the poorer group.

One of the strengths of the RII is that it takes the size of the population across socioeconomic categories into account. In order to compute the RII it is necessary to arrange the sample into the socioeconomic categories. Each group is characterized by a “ridit” which corresponds to the average cumulative frequencies of each of the groups [46]. The RII is computed by regressing ridit scores on the outcome. This makes it possible to adjust for possible confounding by undertaking multivariable analyses. The adjusted models control for the possible confounding effects of sex, age, area of residence, marital status, work status and self-rated health in self-reported unmet need.

We did not conduct a pooled multi-country analysis with a “country” variable for a couple of reasons. First, a large number of countries would have been needed to estimate country effects reliably. Second, the country sample was defined in accordance with the way in which respondents answered questions about oral health services and we were concerned that differences in the distributions of responses (to the oral health questions) in each of the countries would have led to bias in a pooled analysis.

Coefficients were obtained by Poisson models using the logarithmic link function expressed as exp.(β). All statistical analyses were carried out using STATA 13 software (StataCorp, 2013).

## Results

The final country samples of adults aged 50 years and over with complete data on all study variables were 1591 in China, 425 in Ghana, and 1307 in India. See Fig. 1.

### Sample characteristics

The distribution of selected demographic and socioeconomic characteristics in the country samples is given in Table 1. The proportions of females to males were just above 50% in China and India and just under 50% in Ghana. Just over one-third of the adults were aged 50–59 years. Approximately 62% in China and Ghana, and 73% in India were rural residents. Around 20% of the adults belonged to the lowest wealth group in each country. A high proportion had no schooling; just over 50% in India and Ghana, and about 32% in China; about 3% had university level education in all three countries.

**Table 1:** Socio-demographic descriptors of respondents who self-reported oral health problems in year prior, adults aged 50 years and over in China, Ghana, and India, 2007-2010^a^

		China (1591)	Ghana (425)	India (1307)
		n (%)	n (%)	n (%)
Sex	Male	725 (47.85)	218 (54.20)	614 (48.23)
	Female	866 (52.15)	207 (45.80)	693 (51.77)
Age (years)	50–59	508 (34.50)	149 (36.75)	452 (39.74)
	60–69	491 (32.79)	109 (25.04)	455 (32.51)
	70–79	437 (24.54)	111 (25.99)	285 (20.63)
	80+	155 (08.16)	56 (12.22)	115 (07.11)
Residence	Urban	606 (37.73)	162 (37.06)	285 (27.30)
	Rural	985 (62.27)	263 (62.94)	1022 (72.70)
Marital status	Never married	13 (01.08)	3 (00.95)	16 (00.93)
	Married/cohabiting	1241 (80.77)	224 (57.13)	870 (70.93)
	Divorced/separated	337 (18.15)	198 (41.92)	421 (28.14)
Household wealth (quintiles)	1 (Highest)	242 (16.40)	81 (19.24)	273 (22.79)
	2	297 (21.21)	88 (21.59)	253 (17.95)
	3	323 (22.13)	84 (21.37)	260 (20.32)
	4	330 (19.97)	78 (16.86)	270 (18.68)
	5 (Lowest)	399 (20.29)	94 (20.94)	251 (20.26)
Education	University	58 (03.43)	13 (03.20)	36 (03.56)
	Secondary	351 (22.50)	91 (21.56)	190 (17.22)
	Primary	631 (41.93)	81 (20.71)	343 (25.90)
	No-school	551 (32.14)	240 (54.53)	738 (53.33)
Work status	Never worked	126 (06.61)	1 (00.17)	381 (26.83)
	Current worker	681 (45.34)	281 (67.13)	447 (37.73)
	Non-current worker	784 (48.06)	143 (32.70)	479 (35.44)
Self-rated health	Good	377 (25.27)	140 (34.39)	260 (23.10)
	Moderate	715 (43.15)	170 (39.90)	642 (46.01)
	Bad	499 (31.58)	115 (25.71)	405 (30.89)

^a^Data source – WHO Study on global AGEing and adult health (SAGE). [n (%)] Absolute number and weighted percent

### Socio-demographic characteristics and prevalence of self-reported unmet need

Table 2 shows the weighted prevalence of self-reported unmet need for oral health among adults aged 50 years and over in China, Ghana, and India. Prevalence was highest in Ghana (80%) followed by India (62%) and then China (60%). In China the prevalence of unmet need was higher in females (52% versus 48% in males), in Ghana the prevalence was higher in males (55% versus 45% in females) and in India the prevalence was approximately equal for males and females. Unmet need was more prevalent in rural areas in all three countries. In China the prevalence was 71% in rural areas and 29% in urban areas, in Ghana the prevalence was 66% in rural areas and 34% in urban areas, and in India the prevalence was 73% in rural areas and 27% in urban areas.

**Table 2:** Prevalence of self-reported unmet need for oral health services by characteristics, adults aged 50 years and over in China, Ghana, and India, 2007-2010^a^

		China *N* = 1591			Ghana *N* = 425			India *N* = 1307		
Unmet need		n	% (CI)	*p*-value	n	% (CI)	*p*-value	n	% (CI)	*p*-value
	**Total**	927	60.00 (56.18–63.70)		348	80.34 (75.42–84-48)		810	61.97 (56.92–66.77)	
Sex	Male	422	47.79 (43.99–51.61)	0.961	182	55.05 (48.51–61.42)	0.507	395	50.43 (44.41–56.43)	0.2175
	Female	505	52.21 (48.39–56.01)		166	44.95 (38.58–51.49)		415	49.57 (43.57–55.59)	
Age (years)	50–59	242	29.91 (27.24–32.72)	***p*** **< 0.001**	118	34.83 (28.41–41.86)	0.128	237	33.15 (29.10–37.47)	***p*** **< 0.0001**
	60–69	278	32.52 (29.67–35.50)		94	26.73 (21.93–32.14)		283	33.17 (28.58–38.11)	
	70–79	291	26.81 (22.71–31.35)		87	25.11 (20.11–30.86)		202	24.83 (20.62–29.57)	
	80+	116	10.77 (08.11–14.15)		49	13.34 (09.88–17.77)		88	08.85 (06.60–11.78)	
Residence	Urban	276	29.09 (24.83–33.76)	***p*** **< 0.001**	121	34.27 (28.26–40.82)	**0.049**	158	26.72 (17.88–37.90)	0.8069
	Rural	651	70.91 (66.24–75.17)		227	65.73 (59.18–71.74)		652	73.28 (62.10–82.12)	
Marital status	No-married	9	01.28 (00.66–02-48)	**0.031**	1	00.29 (00.01–02.02)	**0.024**	13	01.38 (00.56–03.36)	**0.0250**
	Married	693	78.73 (75.58–81.58)		186	57.58 (51.33–63.74)		521	68.75 (63.87–73.25)	
	Divorce	225	19.98 (17.42–22.82)		161	42.03 (35.87–48.44)		276	29.87 (25.52–34.62)	
Wealth status	1(Highest)	111	13.55 (10.53–17.27)	***p*** **< 0.001**	56	15.59 (11.72–20.45)	**0.021**	152	20.98 (16.91–25.71)	0.6890
	2	150	18.77 (15.33–22.77)		71	21.69 (16.62–27.79)		160	17.84 (14.47–21.79)	
	3	182	22.48 (17.27–28-73)		72	22.71 (17.30–29.21)		156	20.59 (15.00–27.59)	
	4	195	20.28 (16.88–24.16)		66	17.93 (13.69–23.14)		176	19.12 (15.46–23.41)	
	5(Lowest)	289	24.92 (20.90–29.43)		83	22.08 (17.83–27.00)		166	21.47 (16.76–27.07)	
Education	University	18	01.84 (00.95–03.56)	***p*** **< 0.001**	7	01.98 (00.92–04.21)	**0.004**	10	01.53 (00.60–03.84)	**0.0118**
	Secondary	152	18.02 (14.74–21.84)		73	20.89 (16.11–26.64)		104	16.70 (12.56–21.86)	
	Primary	356	40.40 (36.57–44.35)		60	18.55 (13.99–24.17)		195	24.89 (20.70–29.60)	
	No school	401	39.74 (36.29–43.29)		208	58.58 (51.46–65.36)		501	56.89 (49.97–63.55)	
Work status	Never work	91	08.24 (05.45–12.29)	**0.024**	0	0.00	0.217	222	27.14 (22.75–32.03)	**0.0065**
	working	383	45.37 (39.93–50.93)		228	66.82 (60.30–72.75)		253	33.37 (28.44–38.70)	
	Not current	453	46.38 (41.23–51.61)		120	33.18 (27.25–39.70)		335	39.49 (34.57–44.63)	
Self-rated health	Good	199	24.13 (20.58–28.07)	**0.007**	112	33.41 (27.73–39.61)	0.753	147	21.16 (14.58–29.68)	0.4663
	Moderate	399	40.40 (37.01–43.88)		141	40.67 (34.44–47.21)		410	47.66 (42.07–53.30)	
	Bad	329	35.48 (31.57–39.59)		95	25.92 (20.97–31.59)		253	31.18 (25.85–37.06)	

^a^Data source – WHO Study on global AGEing and adult health (SAGE), 2007–2010

[n] Number of respondents who self-reported unmet need for oral health services

[%(CI)] Weighted percent and Confidence Interval

Statistical significance set at *p* < 0.05

All significant estimates are bolded

### Unadjusted logistic regression: Household wealth and self-reported unmet need

Unadjusted logistic regression models for household wealth and education are presented in Table 3. In China, respondents in the poorest household wealth group were significantly almost three times more likely to report unmet need, compared with those in the richest household group (OR 2.85: 95% CI: 1.90–4.27). In Ghana respondents in the poorest household wealth group were significantly three times more likely (OR 2.96: 95% CI: 1.20–7.23) to report unmet need. In India respondents in the poorest household wealth group were 40% more likely to report unmet need (OR 1.44: 95% CI: 0.83–2.50) although this estimate was not statistically significant.

**Table 3:** Unadjusted associations with self-reported unmet need for oral health services, adults aged 50 years and over in China, Ghana, and India, 2007-2010^a^

		China (*N* = 1591) Unadjusted Model		Ghana (*N* = 425) Unadjusted Model		India (*N* = 1307) Unadjusted Model	
		OR (CI)	*p*-value	OR (CI)	*p*-value	OR (CI)	*p*-value
Household wealth (quintiles)	1 (Highest)	1.00		1.00		1.00	
	2	1.15 (0.84–1.58)	0.377	2.24 (1.09–4.63)	**0.029**	1.21 (0.76–1.92)	0.427
	3	1.59 (0.97–2.59)	0.064	3.13 (1.24–7.90)	**0.016**	1.27 (0.76–2.13)	0.361
	4	1.59 (0.97–2.59)	0.064	3.15 (1.43–6.92)	**0.005**	1.31 (0.76–2.26)	0.334
	5 (Lowest)	2.85 (1.90–4.27)	***p*** **< 0.001**	2.96 (1.20–7.23)	**0.019**	1.44 (0.83–2.50)	0.197
Education	University	1.00		1.00		1.00	
	Secondary	1.95 (0.79–4.82)	0.144	3.56 (1.05–12.02)	**0.041**	4.17 (1.20–14.49)	**0.025**
	Primary	2.89 (1.15–7.26)	**0.025**	2.60 (0.82–8.26)	0.104	4.08 (1.15–14.40)	**0.029**
	No school	6.06 (2.27–16.17)	**0.001**	6.39 (2.31–17.71)	***p*** **< 0.001**	5.40 (1.53–19.05)	**0.009**
Sex	Female	1.00		1.00		1.00	
	Male	0.99 (0.77–1.28)	0.961	1.19 (0.71–1.99)	0.507	1.26 (0.87–1.83)	0.218
Age (years)	50–59	1.00		1.00		1.00	
	60–69	1.36 (1.04–1.77)	**0.026**	1.88 (0.97–3.65)	0.060	1.61 (1.10–2.34)	**0.014**
	70–79	1.76 (1.21–2.55)	**0.004**	1.09 (0.58–2.04)	0.797	2.74 (1.85–4.07)	***p*** **< 0.001**
	80+	3.50 (2.10–5.83)	***p*** **< 0.001**	2.22 (0.87–5.67)	0.093	3.15 (1.61–6.16)	**0.001**
Residence	Urban	1.00		1.00		1.00	
	Rural	2.50 (1.85–3.39)	***p*** **< 0.001**	1.80 (0.99–3.26)	0.051	1.08 (0.58–2.01)	0.807
Marital status	Married	1.00		1.00		1.00	
	Divorce	1.38 (1.08–1.77)	**0.012**	0.96 (0.59–1.59)	0.886	1.28 (0.95–1.73)	0.108
	Never married	1.77 (0.62–5.07)	0.280	0.08 (0.01–0.85)	**0.037**	7.05 (1.31–38.12)	**0.023**
Work status	Current worker	1.00		1.00		1.00	
	Non-current	0.92 (0.69–1.21)	0.531	1.11 (0.62–1.97)	0.732	1.84 (1.29–2.61)	**0.001**
	Never worked	1.98 (1.20–3.29)	**0.009**	n/a	n/a	1.39 (0.89–2.16)	0.149
Self-rated health	Good	1.00		1.00		1.00	
	Moderate	0.96 (0.75–1.22)	0.710	1.27 (0.67–2.43)	0.464	1.36 (0.75–2.49)	0.309
	Bad	1.54 (1.17–2.04)	**0.003**	1.20 (0.58–2.47)	0.621	1.27 (0.67–2.40)	0.457

^a^Data source – WHO Study on global AGEing and adult health (SAGE), 2007–2010

[OR (CI)] Unadjusted Odds Ratios and 95% Confidence Intervals

Statistical significance set at p < 0.05 (logistic regression)

[n/a] No result due to no observation in the “never worked” group

All significant estimates are bolded

### Unadjusted logistic regression: Education and self-reported unmet need

The least educated in China were significantly six times more likely to report unmet need (OR 6.06: 95% CI: 2.27–16.17), and over six times more likely in Ghana (OR 6.39: 95% CI: 2.31–17.71). In India the least educated were almost five and a half times more likely to report unmet need (OR 5.40: 95% CI: 1.53–19.05).

### Unadjusted logistic regression: Covariates and self-reported unmet need

Age was significant and inversely associated with self-reported unmet need in China and India, rural residence was significantly associated with unmet need in China as was “bad” self-reported health.

### Adjusted logistic regression: Household wealth and self-reported unmet need

The adjusted logistic regression analysis for household wealth is shown in Table 4. In China, the household wealth variable attenuated to non-significance after controlling for age, sex, area of residence, marital status, work status, and self-rated health. In Ghana however, the association remained significant after controlling for potential confounders; those in the poorest household wealth group were almost three times more likely to report unmet need compared with those in the richest household wealth group (OR 2.84: 95% CI: 1.14–7.11). Household wealth was not significant in India.

**Table 4:** Adjusted logistic regression of household wealth and self-reported unmet need for oral health services, adults aged 50 years and over in China, Ghana, and India, 2007-2010^a^

		China (N = 1591) Adjusted Model		Ghana (N = 425) Adjusted Model		India (*N* = 1307) Adjusted Model	
		OR (CI)	*p*-value	OR (CI)	*p*-value	OR (CI)	*p*-value
Household wealth (quintiles)	1 (Highest)	1.00		1.00		1.00	
	2	0.82 (0.56–1.18)	0.275	2.05 (0.98–4.27)	0.055	1.23 (0.79–1.92)	0.357
	3	1.01 (0.59–1.72)	0.978	2.70 (1.10–6.65)	**0.030**	1.30 (0.76–2.23)	0.335
	4	0.79 (0.46–1.36)	0.338	2.90 (1.32–6.37)	**0.008**	1.40 (0.84–2.34)	0.202
	5 (Lowest)	1.23 (0.80–1.90)	0.340	2.84 (1.14–7.11)	**0.026**	1.65 (0.91–3.00)	0.098
Sex	Female	1.00		1.00		1.00	
	Male	1.04 (0.81–1.34)	0.762	1.39 (0.67–2.87)	0.369	1.61 (1.02–2.54)	**0.042**
Age (years)	50–59	1.00		1.00		1.00	
	60–69	1.24 (0.97–1.58)	0.082	1.75 (0.86–3.60)	0.124	1.48 (1.02–2.14)	**0.038**
	70–79	1.77 (1.29–2.44)	**0.001**	0.97 (0.48–1.97)	0.937	2.52 (1.61–3.95)	***p*** **< 0.001**
	80+	3.41 (1.83–6.35)	***p*** **< 0.001**	1.86 (0.62–5.65)	0.268	2.89 (1.39–5.98)	**0.004**
Residence	Urban	1.00		1.00		1.00	
	Rural	2.99 (2.03–4.42)	***p*** **< 0.001**	1.38 (0.74–2.56)	0.310	1.02 (0.58–1.79)	0.955
Marital status	Married	1.00		1.00		1.00	
	Divorce	0.97 (0.74–1.26)	0.790	1.10 (0.54–2.26)	0.791	1.02 (0.69–1.50)	0.920
	Never married	1.25 (0.26–5.96)	0.777	0.08 (0.01–1.19)	0.067	5.17 (0.86–31.07)	0.073
Work status	Current worker	1.00		1.00		1.00	
	Non-current	1.33 (0.89–1.99)	0.162	1.19 (0.58–2.43)	0.628	1.51 (1.01–2.25)	**0.042**
	Never worked	1.75 (0.99–3.08)	0.052	n/a	n/a	1.75 (1.01–3.05)	**0.049**
Self-rated health	Good	1.00		1.00		1.00	
	Moderate	0.97 (0.73–1.29)	0.850	1.41 (0.71–2.79)	0.321	1.19 (0.69–2.03)	0.529
	Bad	1.23 (0.93–1.63)	0.143	1.02 (0.45–2.28)	0.965	0.89 (0.49–1.60)	0.689

^a^Data source – WHO Study on global AGEing and adult health (SAGE), 2007–2010

[OR (CI)] Adjusted Odds Ratios and 95% Confidence Intervals

Statistical significance set at p < 0.05 (logistic regression)

[n/a] No result due to no observation in the “never worked” group

All significant estimates are bolded

Sex was not significantly associated with self-reported unmet need except in India where males were 60% more likely to report unmet need than females (OR 1.61: 95% CI 1.02–2.54). Sex was not significant in either China, Ghana or India in the unadjusted analyses (See Table 3). In China those in the 80+ year age group were almost three and half times more likely to report unmet need (OR 3.41: 95% CI 1.83–6.35) compared with those in the 50–59 year age group. In India those in the 80+ year age group were almost three times more likely to report unmet need compared with the 50–59 year age group (OR 2.89: 95% CI 1.39–5.98). However, the age gradient was not statistically significant in Ghana. Work status was statistically significant only in India. Compared with current workers, non-current workers were 50% more likely to report unmet need (OR 1.51: 95% CI 1.01–2.25) and those who had never worked were 75% more likely to report unmet need (OR 1.75: 95% CI 1.01–3.05).

### Adjusted logistic regression: Education and self-reported unmet need

The adjusted logistic regression analysis for education is shown in Table 5. In China education attenuated but remained significant, whereby the least educated were three times more likely to report unmet need compared with the most educated (OR 2.87: 95% CI 1.04–7.89) after controlling for age, sex, area of residence, marital status, work status, and self-rated health. In Ghana the odds of unmet need were over eight times higher in the no schooling group compared with the university group after controlling for the confounders, although the wide confidence interval indicates low precision (OR 8.26: 95% CI 2.81–24.31). In India those who identified in the no schooling group were seven times more likely to report unmet need compared with those with university education (OR 7.05: 95% CI 2.14–23.19) after controlling for age, sex, area of residence, marital status, work status, and self-rated health.

**Table 5:** Adjusted logistic regression of education and self-reported unmet need for oral health services, adults aged 50 years and over in China, Ghana, and India, 2007-2010^a^

		China (*N* = 1591) Adjusted Model		Ghana (*N* = 425) Adjusted Model		India (*N* = 1307) Adjusted Model	
		OR (CI)	*p*-value	OR (CI)	*p*-value	OR (CI)	*p*-value
Education	University	1.00		1.00		1.00	
	Secondary	1.59 (0.64–3.95)	0.321	3.86 (1.05–14.15)	**0.042**	4.29 (1.44–12.77)	**0.009**
	Primary	1.70 (0.64–4.49)	0.281	3.44 (1.04–11.35)	**0.042**	4.44 (1.38–14.27)	**0.013**
	No schooling	2.87 (1.04–7.89)	**0.041**	8.26 (2.81–24.31)	***p*** **< 0.001**	7.05 (2.14–23.19)	**0.001**
Sex	Female	1.00		1.00		1.00	
	Male	1.19 (0.91–1.56)	0.209	1.67 (0.79–3.51)	0.178	2.11 (1.27–3.50)	**0.004**
Age (years)	50–59	1.00		1.00		1.00	
	60–69	1.22 (0.92–1.61)	0.171	1.67 (0.84–3.33)	0.141	1.39 (0.96–2.02)	0.083
	70–79	1.53 (1.09–2.15)	**0.015**	0.69 (0.33–1.46)	0.330	2.40 (1.48–3.89)	***p*** **< 0.001**
	80+	2.76 (1.50–5.07)	**0.002**	1.26 (0.41–3.92)	0.685	2.53 (1.20–5.30)	**0.014**
Residence	Urban	1.00		1.00		1.00	
	Rural	2.54 (1.82–3.56)	***p*** **< 0.001**	1.57 (0.85–2.90)	0.152	0.92 (0.53–1.59)	0.768
Marital status	Married	1.00		1.00		1.00	
	Divorce	0.93 (0.71–1.22)	0.597	1.20 (0.59–2.47)	0.608	1.02 (0.70–1.49)	0.908
	Never married	1.18 (0.32–4.44)	0.800	0.08 (0.01–1.64)	0.099	4.78 (0.82–27.88)	0.082
Work status	Current worker	1.00		1.00		1.00	
	Non-current	1.31 (0.88–1.96)	0.179	1.11 (0.55–2.25)	0.767	1.49 (0.99–2.22)	0.053
	Never worked	1.64 (0.92–2.90)	0.091	n/a	n/a	1.69 (1.01–2.83)	**0.047**
Self-rated health	Good	1.00		1.00		1.00	
	Moderate	1.01 (0.75–1.33)	0.992	1.59 (0.82–3.08)	0.172	1.09 (0.63–1.89)	0.749
	Bad	1.23 (0.92–1.64)	0.162	1.21 (0.55–2.65)	0.635	0.86 (0.47–1.57)	0.620

^a^Data source – WHO Study on global AGEing and adult health (SAGE), 2007–2010

[OR (CI)] Adjusted Odds Ratios and 95% Confidence Intervals

Statistical significance set at *p* < 0.05 (logistic regression)

[n/a] No result due to no observation in the “never worked” group

All significant estimates are bolded

In this education model sex was again significantly associated with self-reported unmet need only in India where males were twice as likely to report unmet need as females (OR 2.11: 95% CI 1.27–3.50). In China those who identified in the 80+ year age group were almost three times more likely to report unmet need (OR 2.76: 95% CI 1.50–5.07) compared with those in the 50–59 year age group. In India those in the 80+ year age group were two and a half times more likely to report unmet need compared with the 50–59 year age group (OR 2.53: 95% CI 1.20–5.30). As in the wealth model, the age gradient was not significant in Ghana. Work status was again only statistically significant in India. Compared with current workers, those who had never worked were about 70% more likely to report unmet need (OR 1.69:95% CI 1.01–2.83).

### Unadjusted RIIs: Household wealth and education

Table 6 shows unadjusted RIIs for household wealth and education. In China the relative risks of self-reported unmet need among adults in the poorest household wealth group were significantly 60% higher than those in the richest wealth group (RR 1.61: 95% CI: 1.32–1.96). In Ghana the unadjusted relative risks were significant and 30% higher with the same comparison (RR 1.29: 95% CI: 1.04–1.61). The unadjusted RII was not statistically significant for household wealth in India.

**Table 6:** Unadjusted RIIs for self-reported unmet need for oral health services by household wealth and education, adults aged 50 years and over in China, Ghana, and India, 2007-2010^a^

	China (N = 1591) Unadjusted Model		Ghana (N = 425) Unadjusted Model		India (N = 1307) Unadjusted Model	
	RR (CI)	*p*-value	RR (CI)	*p*-value	RR (CI)	*p*-value
Household wealth	1.61 (1.32–1.96)	***p*** **< 0.001**	1.29 (1.04–1.61)	**0.021**	1.17 (0.92–1.50)	0.209
Education	2.05 (1.65–2.56)	***p*** **< 0.001**	1.33 (1.09–1.61)	**0.004**	1.37 (0.99–1.88)	0.051

^a^Data source – WHO Study on global AGEing and adult health (SAGE), 2007–2010

[RR (CI)] Relative Risk Ratios and 95% Confidence Intervals

Statistical significance set at *p* < 0.05 (Relative Index of Inequality)

All significant estimates are bolded

The unadjusted relative risks of self-reported unmet need in the least educated group were significantly twice that of the highest educated group in China (RR 2.05: 95% CI: 1.65–2.56) and significantly one third higher in Ghana (RR 1.33: 95% CI: 1.09–1.61).

### Adjusted RIIs: Household wealth and education

Table 7 gives adjusted RIIs for household wealth and education, after controlling for possible confounding effects by age, sex, area of residence, marital status, work status, and self-rated health. In this adjusted analysis the RII for household wealth was statistically significant only in Ghana (*p* < 0.05) where the relative risk of unmet need among adults in the poorest compared with the richest household wealth groups was almost 30% higher (RR 1.28: 95% CI 1.04–1.56).

**Table 7:** Adjusted RIIs for self-reported unmet need for oral health services by household wealth and education, adults aged 50 years and over in China, Ghana, and India, 2007-2010^a^

	China (N = 1591) Adjusted Model		Ghana (*N* = 425) Adjusted Model		India (N = 1307) Adjusted Model	
	RR (CI)	*p*-value	RR (CI)	*p*-value	RR (CI)	*p*-value
Household wealth	1.09 (0.89–1.32)	0.401	1.28 (1.04–1.56)	**0.018**	1.23 (0.97–1.57)	0.089
Education	1.49 (1.18–1.88)	**0.001**	1.37 (1.12–1.67)	**0.002**	1.53 (1.18–1.97)	**0.001**

^a^Data source – WHO Study on global AGEing and adult health (SAGE), 2007–2010

[RR (CI)] Adjusted Relative Risk Ratios and 95% Confidence Intervals

Statistical significance set at *p* < 0.05 (Relative Index of Inequality)

All significant estimates are bolded

The adjusted relative risks of self-reported unmet need among the least educated compared with the highest educated group, were significantly almost 50% higher (RR 1.49: 95% CI: 1.18–1.88) in China, significantly almost 40% higher in Ghana (RR 1.37: 95% CI: 1.12–1.67) and just over 50% higher in India (RR 1.53: 95% CI: 1.18–1.97).

Comparing the adjusted and unadjusted models, we see positive attenuation in China and negative attenuation in India. The net effects of older age, rural residence, never having worked and having bad self-rated health contributed to the education inequality in China, (Table 3). However, in India the net effects of the confounders offset the inequality because when they are held constant (in the adjusted analysis) the inequality increased to significance. This is also attributed to interaction between sex and education in the India sample. Men were generally more highly educated than women, and education is negatively associated unmet need, yet men were more likely to report unmet need for oral health care. In India therefore, association between education and unmet need was modified by sex.

## Discussion

The prevalence of self-reported unmet need was 60%, 80% and 62% in China, Ghana, and India respectively, which is consistent with studies that include younger populations. An analysis of data collected (from adults aged 18 and over) in the World Health Surveys (2002–2004) reported unmet need for oral health services in China at 56%, Ghana 68%, and India 52% [6]. The proportions of adults aged 65–74 years with unmet need for oral health care have been estimated at 70% in the African region and 60% in the Asian region [47].

When adjusted for age, sex, area of residence, marital status, work status and self-rated health, the estimated education RIIs in China and India were similarly high (1.49 and 1.53 respectively) and significant (*p* < 0.01). The lower education RII was in the poorest of the three countries, Ghana (1.37). However, this result may underestimate “true” unmet need in Ghana where many older, poorer and less educated people may be unaware of their oral health service needs. The finding that higher education was protective of unmet need for oral health care in China and India is consistent with what is known about the take-up of health promotion messages by more highly educated groups [48, 49]. China has introduced awareness of oral health care and illness prevention through mass media programs such as “National Teeth Day” [30, 50]. A recent review of oral public health in India concluded that priorities should be directed towards preventive oral health care by strengthening oral health education and combining oral health programs with general health care programs [51].

In China, household wealth was protective of unmet need for oral health services only in the unadjusted regression, suggesting that the confounders contributed to the wealth inequality. In China oral health care is delivered by a large government controlled public sector with over 85% of the total expenses covered by patients’ out-of-pocket payments. In recent years the numbers of dentists and oral health institutions in China have increased although oral health services are not being utilised efficiently [30]. It is important that oral health services target specific population sub-groups, such as those who are older, sicker, less educated and living in rural areas.

The Chinese health care insurance system is undergoing major reforms that are intended to improve access to affordable health care for all and alleviate inequalities in access to care that exist for rural residents, low income households and older adults [52]. Medical insurance for oral health, including preventive services is a part of the policy mix. However regardless of improvements in the average health status of the population in recent decades, socio-demographic factors contribute to inequalities in physical and oral health [30].

The RIIs for education and household wealth in Ghana were consistent across the adjusted and unadjusted models. Ghana’s government has only recently begun to prioritise NCDs as an emerging public health threat because the country’s health burden still includes infectious communicable conditions. A study of NCDs in adults aged 50 and over in Ghana found that 45% reported having oral health problems compared with 33% who reported hypertension [53]. In Ghana there are major medical workforce shortages with oral health no exception. Older poorer adults are a vulnerable segment of the population [33]. Many older people in Ghana live in poverty without access to social services or pensions and as a consequence, face barriers in accessing and using oral and general health care [35].

The study results did not show significant association between household wealth and unmet need in India. On one hand this may be surprising because under India’s privately controlled health sector, health care is not affordable for a large segment of the population [54, 55]. Yet this can be explained in part by factors that were not included in the models, for example social and cultural norms and practices. In India there is widespread acceptance of self-medication for oral problems and a common view that tooth loss is a natural and acceptable part of the ageing process that does not require clinical intervention [51]. This is compatible with the view in India that oral health is the responsibility of the individual rather than the state. A national assessment of oral health care in India from 2004 to 2014 which identified neglect in public health policy and government responsibility also acknowledged that in addition to socio-economic factors, customs, cultural values and attitudes impact on oral health [37].

In the Indian sample the RII for education was not statistically significant in the unadjusted model, but was significant (*p* < 0.001) in the presence of age, sex, area of residence, marital status, work status, and self-rated health. This can be explained by the interaction between sex and education with men more highly educated than women, but also reporting more unmet need. This is indicative of gender issues in a country in which there is still much discrimination against women [56]. Males are valued more highly and this impacts on the reporting of health needs. In addition, oral health policy making in India is fraught with challenges that include enhancing primary oral health care, breaking down taboos and myths about oral hygiene and developing tailored education programs that take into account the diversity of ethnic groups and their social and geographic distribution across a very large and widespread population.

### Strengths and limitations

The study has a number of strengths. In addition to extending the public health literature on oral health inequalities, the analyses contribute evidence of self-reported unmet need for oral health care by adults aged 50 and over in three contrasting LMICs. The data were captured and measured using validated WHO survey instruments. Survey questions pertaining to self-reported unmet need are consistent with those used by other international studies including the Survey on Health, Ageing and Retirement and the European Union Survey of Income and Living Conditions (22). Household wealth, as measured here using principal component analysis, is stable indicator of socioeconomic position in LMICs [57]. A further strength is the use of the RII which is a recognised robust measure of socioeconomic status in populations. Finally, given the global debates about universal health coverage and financing, these results can provide a basis for discussion about the need for publicly funded oral health services [58].

However, there are also some limitations. This was a cross-sectional analysis and causation cannot be claimed. Although WHO-SAGE used a stratified multi-stage sampling design to ensure that the country samples were representative of their national populations, the country samples were conditioned according to responses to two particular questions on oral health. We therefore acknowledge the possibility of sampling bias in the way in which unmet need was interpreted by respondents. The poor for example, may not be aware of their oral health needs because they have more important priorities such as obtaining and providing sufficient food and clean water for their families. Although our analyses showed for example that women in India reported less unmet need than men, we cannot infer from this that women’s oral health needs were met.

The analyses focused on curative oral health services in older adults. This was necessary because of data availability however we acknowledge that there is also the need for preventive interventions and services and that oral health applies across all age groups. This topic, as well as gender issues mentioned above, could be the subject of future research using qualitative as well as quantitative methodologies.

There are many factors associated with the measurement of oral health in LMICs and we were unable to cover them all. Psychosocial determinants such as social networks, social capital, self-efficacy, the environment, cultural attitudes and perceptions and also structural factors including physical infrastructure and supply of health services, medical insurance and distance to available oral health facilities, are some areas of follow up for future research.

### Policy implications

The numbers of older adults will continue to increase in all parts of the world and LMICs are no exception [3]. The findings suggest questions for policy makers such as: How will unmet need for oral health care be met? What strategies are currently available? Given what is known about the links between NCDs, risk factors and oral health, what possible joint contributions can oral health and public health professionals make? [59]. How will preventive services and oral health education be integrated with curative services? How can taboos and myths about oral health be changed?

This study establishes the importance of education in seeking oral health care and supports the case for integrating oral health into general health promotion and education programmes in LMICs. Lessons should be learnt from the HICs where the take-up of health promotion messages has been greater among the more highly educated. New ways of targeting less well educated groups in LMICs with health promotion messages, are needed.

There is now more than ever a need to integrate oral health into public health policies [7, 60, 61]. New policies must be backed by sufficient human and other resources so that unmet need can be firstly recognised, and secondly, met through skilled service providers. The three countries in this study are all at different stages of development with regard to public oral health and they all face different challenges with regard to identifying and meeting the needs of their populations. Resources must be committed to oral health care infrastructure, education, data collection, the training and location of oral health professionals and oral health promotion. The goals of universal health coverage must explicitly include oral health. Political commitment and support is needed in all countries. This resonates with issues raised in the literature regarding the need for serious policy commitment to dentistry and oral health worldwide [62].

## Conclusions

The findings show that educational attainment is protective of unmet need for oral health services in three countries at different levels of social and economic development. Clearly more oral health needs can be met with increased supply of oral health professionals – trained with awareness about inequalities - implementing appropriate targeted education programs directed at all socioeconomic groups, regardless of wealth and education. Universally oral health is a public health issue requiring attention and action on multiple levels and across the public private divide.

## Abbreviations

Cis: Confidence intervals
FDI: Fédération dentaire internationale
HICs: High-income countries
LICs: Low-income countries
LMICs: Low- and middle-income countries
NCDs: Non-communicable diseases
ORs: Odds ratios
RII: Relative index of inequality
SAGE: Study on global AGEing and adult health
WHO: World Health Organization
